# Appearance-related concerns in individuals with pathological skin picking—a comparison with individuals with dermatological conditions and skin-healthy controls

**DOI:** 10.3389/fmed.2023.1075743

**Published:** 2023-05-03

**Authors:** Jennifer Schmidt, Christina Gallinat, Alexandra Martin

**Affiliations:** ^1^Münster Department of Health, FH Münster—University of Applied Sciences, Münster, Germany; ^2^Center for Psychotherapy Research, University Hospital Heidelberg, Heidelberg, Germany; ^3^Clinical Psychology and Psychotherapy, School of Human and Social Sciences, University of Wuppertal, Wuppertal, Germany

**Keywords:** pathological skin picking, skin picking disorder, excoriation disorder, body dysmorphic disorders, body image, body dissatisfaction, mental health, skin diseases

## Abstract

**Introduction:**

Pathological skin picking (PSP) is an excessive behavior which characterizes Skin Picking Disorder. Individuals repeatedly pick their skin and cause skin lesions, but are unable to control the behavior, which can cause severe distress. Visible self-inflicted skin lesions can additionally affect individuals with PSP due to emerging appearance-related concerns. However, these concerns and their role in PSP have hardly been studied, especially not in comparison with individuals with dermatological conditions and skin-healthy controls.

**Methods:**

The present cross-sectional study (*n* = 453, 83.9% female, 15.9% male, 0.2% diverse) aimed at analyzing appearance-related concerns and mental health outcomes between four groups: Individuals with PSP and dermatological conditions (SP/DC; *n* = 83), PSP without dermatological conditions (SP; *n* = 56), dermatological conditions without PSP (DC; *n* = 176) and skin-healthy controls (SH, *n* = 138). We compared questionnaire data on dysmorphic concerns, appearance-based rejection sensitivity, and body dysmorphic symptoms, as well as PSP-symptoms and mental health outcomes (depression, anxiety, and self-esteem) between groups.

**Results:**

The analyses showed a significant multivariate group effect in the appearance-related variables, *F*(6, 896) = 19.92, Wilks’ *Λ* = 0.78, *p* < 0.001, and mental health outcomes, *F*(6, 896) = 16.24, Wilks’ *Λ* = 0.81, *p* < 0.001. The SP/DC group had the strongest appearance-related concerns and mental health impairments, followed by the SP group, the DC group and the SH group. The SP/DC group and SP group only differed significantly with regard to dysmorphic concerns, but not in other variables. The DC group was less affected but still showed higher dysmorphic concerns and mental health impairments than skin-healthy controls. In contrast to the PSP groups, the other two groups did not exceed clinically relevant cut-off scores.

**Discussion:**

The present study shows that individuals with PSP exhibit strong appearance-related concerns, regardless of the presence or absence of underlying or comorbid dermatological conditions. These findings shed new light on the importance of appearance-related concerns in Skin Picking Disorder and the role of PSP as a potentially overlooked risk factor in dermatological patients. Therefore, appearance-related concerns should be explicitly addressed in dermatological and psychotherapeutic settings. Future studies should also include longitudinal and experimental analyses to more clearly classify the role of appearance-related concerns in the etiology of PSP and Skin Picking Disorder.

## 1. Introduction

With regard to pimples, crusts or other skin imperfections, it is a common cosmetic routine for most people to remove these imperfections by picking, squeezing, or scratching. A large proportion of the population generally reports engaging in this skin picking behavior on an occasional or regular basis, for example, 46.1% in a Polish sample of young adults (1), 62.7% in a US community sample (2), and 91.7% in a German student sample (3).

However, for some people the extent of skin picking clearly exceeds cosmetic routine and becomes a clinically relevant behavior referred to as pathological skin picking (PSP). PSP represents the core symptom of a mental disorder, which was first included as a separate diagnosis in the Diagnostic and Statistical Manual of Mental Disorders (DSM-5) in 2013. Excoriation (Skin Picking) Disorder (SPD) is a mental disorder in which individuals repeatedly and pathologically pick their skin (i.e., PSP), resulting in skin lesions and tissue damage. Despite frequent intentions to reduce or stop the behavior, affected individuals do not manage to refrain from PSP and clearly experience distress and social impairments. These impairments arise from the feeling of loss of control but also from the frequently visible consequences of skin picking, like wounds, inflammations and scars. PSP in the context of SPD must not be explained by substance influences, medical conditions or other psychological disorders [e.g., body dysmorphic disorder (BDD)] (4).

While the DSM-5 classifies SPD as a disorder within the obsessive–compulsive spectrum, the current International Classification of Diseases-11 further highlights its character in a subcategory of body-focused repetitive behavior disorders, together with other related disorders, such as pathological hair pulling (trichotillomania) or a residual category including nail biting (onychophagia) or cheek biting [e.g., (5–7)].

The reported prevalence rates of SPD vary depending on the respective diagnostic assessment and sample. A recent study by Grant and Chamberlain (8) reported a current prevalence of 2.1% with SPD and a lifetime prevalence of 3.8% in a large community sample, while the DSM-5 reports a lifetime prevalence of 1.4% (4). Most studies find a higher prevalence of SPD among women compared to men [e.g., (9, 10)], and current comorbidities include generalized anxiety disorder (63.4%), depression (53.1%), panic disorder (27.7%), post-traumatic stress disorder (27.2%), obsessive–compulsive disorder (26.3%), attention-deficit hyperactivity disorder (23.5%), eating disorders (19.3%), drug or alcohol abuse (16.0%), trichotillomania (12.7%), bipolar disorder (12.2%), and tic disorder (7.0%) (8).

Frequently mentioned triggers of PSP in SPD, are confrontations with skin imperfections, like pimples, blackheads, scabs, pustules, or crusts. Many affected individuals report having difficulties suppressing the behavior when confronted with these skin imperfections (11). Therefore, transient or persistent skin conditions may increase the risk for PSP (12–14). Subsequently, PSP may occur in individuals with transient (e.g., pubertal) skin conditions and persist as a behavior even after the skin conditions have vanished in adulthood. Similarly, PSP may develop in individuals with long-standing dermatological conditions (e.g., acne, atopic dermatitis, and psoriasis) and persist in a distressing manner over time. Especially in these groups of persons the additional problem of PSP besides the actual dermatological diagnosis can easily be overlooked (13).

Furthermore, distress and states of emotional tension and insufficient abilities in emotion regulation often lead to skin picking to relieve internal stress (15, 16). Those affected often report a trance-like state for the duration of the skin manipulations, in which time is sometimes forgotten and dissociative states occur (17). While PSP often leads to short-term relaxation and stress reduction, the repeated episodes elicit feelings of shame and guilt in the long term (16–18). In addition to the stressful experience that it is difficult to stop skin picking and to experience a lack of understanding from their social environment as well as from health care professionals [e.g., dermatologists; (18–20)], those affected often also suffer from the visible consequences of the behavior, for example scabs or scars. To avoid skin blemishes or to cover or treat skin picking wounds, many affected people undergo various cosmetic procedures (e.g., dermabrasion, laser therapy) and use camouflaging make-up (21, 22).

These treatments and camouflaging procedures can, in turn, compromise wound healing or cause further skin blemishes, which may then trigger further skin picking episodes. This often results in significant scarring, which is often distressing to those affected. As reported in clinical reports and in the general literature on SPD, many individuals with SPD therefore suffer from the self-perceived disfigurement caused by their own behavior (21). However, the actual empirical data on this relationship is still scarce. Still, a recent study by Gallinat et al. (23) showed specific evidence that SPD is associated with a negative body image.

Reports of cooccurring skin picking behaviors and BDD (24, 25) further illustrate possible relations of dysmorphic concerns and PSP. Fear of being rejected by others because of one’s appearance due to the clearly visible skin imperfections [i.e., appearance-based rejection sensitivity (ARS); (26)] may increase over the course of the mental illness and additionally contribute to avoidance behaviors, social withdrawal, decreased self-esteem, and comorbid anxiety and depression (27–29). In line with this assumption, Tucker et al. reported that a large proportion of individuals with PSP show social withdrawal, or avoid social events and going into public (18). However, to date there are only few empirical reports on the relation of these avoidance behaviors with appearance concerns and its role in individuals with PSP has not been examined.

The study on body image and PSP by Gallinat et al. (23) reported mainly correlative associations between PSP and body image disturbances. In addition, the study did not include comparisons with skin-healthy controls or other control groups that might be affected by a similar skin appearance. Here, potential groups of interest include individuals with (visible) dermatological conditions (e.g., acne, atopic dermatitis or psoriasis) that might also promote skin picking behavior, PSP and/or body image concerns (12, 14, 30–32).

The present study is therefore addressing the research question to what extent individuals with PSP with (SP/DC) and without (SP) dermatological conditions differ from individuals with dermatological conditions only (DC) and skin-healthy controls (SH) regarding the degree of their dysmorphic concerns, ARS, and BDD-symptoms. Here, we focus on PSP as a pathological behavior rather than the full syndrome of SPD, which would require a clinical diagnosis and exclusion of differential diagnoses. As further variables, we will also examine group differences in general mental health outcomes (i.e., depression, anxiety, and self-esteem). We hypothesize that individuals with PSP (with or without dermatologic conditions: SP/DC and SP) have more pronounced appearance concerns than both participant groups without PSP (DC and SH).

## 2. Materials and methods

### 2.1. Study design

The study had a cross-sectional design and was conducted as an online survey using SoSci-Survey software (33). Data collection took place in a German convenience sample in spring to summer 2018. All participants provided active informed consent via the online form of the questionnaire. The study was approved by the Ethics Committee of the University of Wuppertal and adhered to the Helsinki Declaration. In addition to answering the research questions presented here, the study also pursued the purpose of validating newly developed translated measurement instruments from the field of skin picking research. Therefore, the number of measurement instruments used in the study was greater than the number of measures presented here and study participation took approximately 30 min. Participants could be notified of study results via email upon request. Students at the University of Wuppertal were able to receive course credit for participation. For participants outside of the university, the allowance consisted of the opportunity to win a gift certificate worth 10 Euros.

### 2.2. Participants

We recruited participants via newsletter announcements and flyers at the University of Wuppertal, websites of psychological journals, flyers in dermatological practices and via various social media platforms. Individuals with PSP were recruited specifically via the newsletter and the internet-forum of the German Self-Help Network for Skin Picking, as well as via Facebook groups on the topic of skin picking. Overall inclusion criteria were legal age in Germany (18 years or above) and sufficient German language skills to understand the questionnaire. There were no general exclusion criteria for the study participation.

However, additional criteria were established for grouping the four groups of interest: (1) skin-healthy individuals without skin picking or dermatological conditions (SH), (2) dermatological conditions without PSP (DC), (3) PSP without skin conditions (SP), and (4) individuals with dermatological conditions and PSP (SP/DC).

For the definition of dermatological conditions to be considered, we decided to focus on three common dermatological conditions that are usually associated with visible skin irregularities and for which previous studies have already demonstrated possible impairments in mental health and psychosocial impairments (34–38). These included acne, atopic dermatitis, and psoriasis. To be included under the dermatological condition subgroups, participants had to indicate that they had ever received a medical diagnosis (lifetime diagnosis) of one of these three dermatological conditions. Individuals who reported other dermatological conditions (e.g., vitiligo, urticaria, rosacea, warts, and alopecia etc.) were excluded from this analysis.

With regard to the PSP subgroups, participants had to be recruited via calls in the German Self-Help Network for Skin Picking and Facebook groups via a separate recruitment link and had to report values >7 on the German version of the Skin Picking Impact Scale [SPIS-D; (39)] to indicate PSP instead of subclinical skin picking. This cut-off corresponds to the original English SPIS (40) and was applied to assure a clinically relevant severity of the PSP at the time of the study. Here, subgrouping into the SP/DC and SP groups was dependent on the presence or absence of a diagnosis of one of the aforementioned skin conditions.

To be classified in the groups without dermatological conditions (SH or SP), participants had to indicate that they have never received a medical diagnosis of any dermatological condition. For the skin-healthy group, participants further had to indicate that they are currently not affected by any skin condition and have a score ≤7 on the SPIS-D.

After exclusion of unsuitable datasets that met exclusion criteria or did not fulfil quality or classification requirements (see Section 2.3.6.1), *n* = 453 participants remained, leading to *n* = 138 participants for the SH group, *n* = 176 for the DC group, *n* = 56 for the SP group, and *n* = 83 for the SP/DC group.

An *a priori* power analysis with G*Power 3.1.9.7 (41) indicated that a sample of 336 participants would be sufficient to detect medium effects between the four groups on eight response variables in a multivariate analysis of variance (MANOVA) with a conservative *α* error of 0.0001 due to multiple comparisons and a statistical power 1-β of 0.95. Thus, the present sample size was determined to be sufficient for the planned analyses.

### 2.3. Assessment instruments

#### 2.3.1. Sociodemographic data

To describe the sociodemographic characteristics of the sample, we recorded age, gender (male/female/other), highest school degree, highest professional degree, and current employment (yes/no).

#### 2.3.2. Dermatological conditions

Dermatological conditions were assessed via self-report. First, we asked participants if they had ever been diagnosed with a skin condition (yes/no). Afterwards, the participants had the opportunity to select from a list of different skin conditions those which they had been medically diagnosed with [e.g., allergies, fungal infections, atopic dermatitis, seborrheic eschar, rosacea, psoriasis, (stages of) skin cancer, alopecia areata, herpes zoster, other herpes diseases of the skin, warts, pruritus, eczema, contact allergies, chafing skin or others with a free entry option].

#### 2.3.3. Assessment of skin picking

Skin picking behavior, severity, and corresponding impairments were assessed using the modified German translation of the Skin Picking Scale-revised (mSPS-D) and the German Skin Picking Impact Scale (SPIS-D) (38). The latter tool was used as a screening instrument for group assignments.

The mSPS-D assesses the frequency, intensity, and ability to control skin picking urges on nine items with five-point Likert scales (e.g., 0 = no urge, 4 = ongoing urge [>8 h per day]). The wording of the answer options is adapted to the wording of the items and therefore varies (38). Compared to the original Skin Picking Scale-Revised (42), the mSPS-D includes one more item because the original item „How much control do you have over your skin picking? To what degree can you stop yourself from picking? “was divided in two items in line with other instruments to assess BFRBs, such as the Massachusetts General Hospital Hairpulling Scale (43). In addition, the wording of items and answer options has been shortened to make the instrument more time economic (39). Higher sum scores indicate greater symptom severity and impairment from skin picking. The mSPS-D has demonstrated good psychometric properties in a validation study (39). The internal consistency in the present sample was excellent (*α* = 0.95).

The SPIS-D (39) is based on the English Skin Picking Impact Scale (40) and captures impairments due to skin picking in various life domains (e.g., relationships, shame, daily routines) on 10 items. Answers are provided on five-point-Likert scales (0 = not at all; 4 = severe). The SPIS served as a screening instrument to classify PSP, using the cut-off score >7 suggested by Keuthen et al. (40). The answer format of the German adaptation differs slightly from the original scale which is rated on 6-point-Likert scales. Although this lowers the total achievable score of the German version, we assume that the screening cut-off value proposed by Keuthen et al. (40, see also: 44) is still sufficiently sensitive as a conservative measure to identify individuals with PSP. The internal consistency in the present sample was excellent (*α* = 0.97).

#### 2.3.4. Appearance concerns

As primary outcomes, appearance-related concerns were assessed using three different measurement instruments in German to capture different relevant facets of appearance-related concern: The Dysmorphic Concerns Questionnaire (DCQ), the Appearance-based Rejection Sensitivity Scale (ARS-D), and a brief screening for symptoms of BDD, based on the DSM-5 diagnostic criteria (BDD-screen).

##### 2.3.4.1. Dysmorphic Concerns Questionnaire

Dysmorphic concerns were assessed using the German translation of the DCQ (45) as an economic and widely used screening instrument in clinical settings. It consists of seven items by which respondents report their appearance-related worries and behaviors compared to the scale of most other people (e.g., “Have you ever worried about a particular aspect of your appearance?”) with a four-point Likert-scale to provide answers from 0 = not at all to 3 = much more than other people. The sum score ranges from 0 to 21 with higher overall scores indicating stronger dysmorphic concerns. The unidimensional scale has been shown to have good psychometric properties (46), and its sum score is frequently used for identification of cases with clinically relevant dysmorphic concerns, for example, using a cut-off of ≥9 in community samples (47), or a more conservative score of ≥11 in samples with dermatological conditions (45). The internal consistency in the present sample was good (*α* = 0.86).

##### 2.3.4.2. Appearance-based Rejection Sensitivity Scale

As an interpersonal aspect of appearance-based concerns, ARS (26) was assessed using the German ARS-D (48). The questionnaire (short-version) consists of 12 items assessing specific appearance-related scenarios in terms of the extent to which these scenarios generate worry about being rejected on the basis of appearance (a): affective component; response format ranging from 1 = very unconcerned to 6 = very concerned and how likely rejection experiences are rated in these scenarios (b): cognitive component; response format ranging from 1 = very unlikely to 6 = very likely. An example scenario would be “You are at a dance and all your friends have been asked to dance, except you”.

Answer scores for affective and cognitive components of each item are first multiplied and then summed up for an overall score. Higher values indicate higher ARS. The instrument has shown good psychometric properties and discriminative validity to differentiate between groups with and without clinically relevant appearance concerns (48). The internal consistency in the present sample was excellent (*α* = 0.94).

##### 2.3.4.3. Screening for body dysmorphic disorder

The adapted short version of the BDD screening (49) consists of four items that assess core criteria of BDD according to the DSM. Item 1 assesses the belief of having ugly or disfiguring body features “Do you think you have one or more ugly or disfigured body parts although other people do not share this opinion or believe your concern to be markedly exaggerated?” (yes/no); Item 2 assesses the individual suffering due to the preoccupation with these body features “Is the preoccupation about the ugly or disfigured body parts very distressing to you?” (yes/no); Item 3 assesses impairments due to the preoccupation with these body features “Are you so affected by concerns about your own physical disfigurement that it impacts your daily life (e.g., at work, in relationships with others)?” (yes/no); and item 4 asks about the duration (years) since when the worries and preoccupation about the respective body features occur. With confirmation of items 1– 3 participants were classified as *BDD positive* cases. Otherwise, they were classified as *BDD negative* for the present analyses. Here, we aimed at comparing the proportions of positive screenings between the groups.

#### 2.3.5. Assessment of mental health variables and self-esteem

As secondary outcomes, we assessed mental health variables to compare additional possible mental health impairments that might result from or be associated with dermatological conditions and/or skin picking. Here, we focused on symptoms of depression and of anxiety, and general self-esteem.

##### 2.3.5.1. Patient Health Questionnaire-9 for depression

Depressive symptoms were assessed with the German version of the Patient Health Questionnaire-9 (PHQ-9) (50), a frequently used screener for symptoms of major depression according to the DSM-5 criteria. The questionnaire assesses the frequency of depressive symptoms within the last 2 weeks on nine items with 4-point Likert-scales ranging from 0 = not at all to 3 = nearly every day. Higher sum scores (range 0–27) indicate more severe depressive symptoms. Good psychometric properties have been reported for the German version (51). For the present study, internal consistency was good (*α* = 0.89).

##### 2.3.5.2. General Anxiety Disorder Scale-7

We assessed symptoms of anxiety with the German General Anxiety Disorder Scale-7 (GAD-7), the anxiety form of the Patient Health Questionnaire (52). The scale consists of seven items that assess the presence of anxiety symptoms within the last 2 weeks on 4-point Likert scales, ranging from 0 = not at all to 3 = nearly every day. The symptoms include for example, worries, nervousness/tension, difficulties to relax etc. Higher sum scores indicate more severe anxiety symptoms. For the present study, internal consistency was excellent (*α* = 0.90).

##### 2.3.5.3. Rosenberg Self-Esteem Scale

We measured participants’ general self-esteem with the German Rosenberg Self Esteem Scale (RSES) (53). The self-report scale captures self-esteem as a trait on 10 statement-items with four-point Likert scales (0 = strongly disagree; 3 = strongly agree). Inverted items have to be recoded before an overall sum score is calculated. Higher values in this sum score indicate stronger self-esteem. Good psychometric properties have been reported for the RSES (53), and the internal consistency in the present study was excellent (*α* = 0.92).

#### 2.3.6. Procedure

For participation, the online questionnaire was accessible via a hyperlink sent with the study calls. Participants thus reached the study’s information page, on which participation requirements, the topic and duration of the survey, as well as the research ethics aspects of voluntary participation, the possibility of withdrawal without disadvantages, and the anonymization of the data were explained. In addition, the contact details of the researchers for queries were listed on the page.

Then, the interested respondents were directed to the consent form page and indicated that they had read, understood, and agreed with the terms and conditions of participation and were at least 18 years old. If respondents did not consent here, the survey was automatically terminated.

The survey began with questions about sociodemographic data. Subsequently, questions were asked about the dermatological diagnoses and current impairments, followed by the questionnaires on skin picking. Afterwards, participants filled in the ARS-D, RSES, DCQ, GAD-7, and PHQ-9, as well as additional questionnaires that were target variables for another research question (e.g., on former teasing experiences, eating behavior).

The progress of the survey was displayed with a visual progress bar on every page. In general, except for the informed consent, participants were able to skip single questions or pages in case they did not want to answer them. However, in case of blank answers, a warning pop-up asked the participants, whether they want to add answers for the missing items. Upon completion of the survey, participants were thanked and given information on how to enter the raffle or receive course credit.

##### 2.3.6.1. Data analysis

Overall, a total of *N* = 765 individuals completed the questionnaire, whereof *n* = 11 participants had to be excluded due to an age <18 and *n* = 24 participants had to be excluded for insufficient data quality, because they showed conspicuously fast completion behavior indicated by quality indicators of the questionnaire software (DEG_TIME values >100) (54). Participants who could not be assigned to any of the four groups of interest (DC/SP, SP, DC, and SH), were excluded from the analyses (*n* = 300) and one person had to be excluded after the grouping process because she indicated both, to have never been diagnosed with a skin condition and to have been diagnosed with acne (*n* = 1), leading to a final analysis sample of *n* = 453. Single missing values in questionnaires were replaced by means of multiple imputation technique (*m* = 20), selecting the imputation with the least deviations from the mean values in the original dataset, which showed only a very small deviation of 0.02 points at maximum.

We calculated descriptive statistics for the sociodemographic characteristics and skin conditions, frequencies and proportions of positive BDD screenings as well as means and standard deviations for the scales on appearance-concerns, skin picking, and mental health. After checking for violations of relevant assumptions, we used three multivariate analyses of variance (MANOVAs) with Wilks Tests to assess appearance concerns (DCQ, ARS-D), and mental health impairments (PHQ-9, GAD-7). Significant multivariate effects were followed up by analyses of variance (ANOVAs) with *post hoc*-comparisons to assess significant differences between the four groups. Additional ANOVAs were conducted for the assessments of differences in skin picking symptoms (mSPS-D) and self-esteem (RSES).

In case of violations of the normality assumption, the results of the ANOVAs were compared to those of a Kruskal-Wallis-test but there were no deviations in results. Thus, ANOVA results are reported throughout the manuscript. In case of violations of the assumption of homogeneity of variances, the results of Welch-ANOVA and of the Games-Howell *post-hoc* tests are reported. Finally, we conducted a *χ*^2^-test with exact Fisher-test to assess different proportions of positive BDD screening between the groups.

The significance level for the analyses was set to *p* < 0.05. All statistical analyses were performed using IBM SPSS 28 and JASP (55).

## 3. Results

### 3.1. Sample characteristics

The analysis sample consisted primarily of women (83.9%) in middle adulthood (M = 30.20 years, SD = 10.54 years) with participants’ ages ranging between 18 and 68 years. The level of education can generally be described as high, as the majority (74.0%) of the sample had a high school diploma as their highest school qualification. Just under one-third of the sample was currently in a degree program or vocational training, while over 40% had already attained at least a bachelor’s degree or higher. Two-thirds of the participants were currently employed (full- or part-time). Among the groups with diagnosed dermatological conditions, the majority (56.0%) reported diagnosed acne, while 46.4% were affected by atopic dermatitis. Psoriasis was the least common diagnosed dermatological condition, accounting for only 8.1% (multiple answers were possible).

Overall, the groups differed significantly in age and sex, with *post hoc* tests showing that the difference was only notable between the DC group and the SP group. The latter was younger, but the group difference was no longer significant in the Bonferroni-corrected *post hoc* test (*p* = 0.059). With regard to gender differences, the proportion of female participants was significantly higher in the two groups with PSP (SP/DC and SP). Therefore, all ANOVAs were repeated with age and gender as covariates in additional analyses. Detailed information on the sample characteristics are displayed in Table 1.

**Table 1:** Sample characteristics of the subgroups and the overall sample.

Variable	SH	DC	SP	SP/DC	Total	Test statistics
*n*	138	176	56	83		
Age *M* (SD) [Range]	29.49 (11.11) [18–68]	31.85 (10.98) [19–66]	27.60 (9.42) [18-61]	29.12 (8.78) [18-62]	30.10 (10.54) [18–68]	***F*(3,449) = 3.04, *p* = 0.029, *η***^**2**^ **= 0.020**
**Gender *n* (%)**						***Χ***^**2**^**(6) = 30.58, *p* < 0.001, *C* = 0.25**
Female	99 (71.7)	148 (84.1)	53 (94.6)	80 (96.4)	380 (83.9)	
Male	38 (27.5)	28 (15.9)	3 (5.4)	3 (3.6)	72 (15.9)	
Other	1 (0.7)	0 (0.0)	0 (0.0)	0 (0.0)	1 (0.2)	
**School degree *n* (%)**						***Χ***^**2**^**(12) = 25.24, *p* = 0.014, *C* = 0.23**
Secondary/elementary school diploma	2 (1.4)	4 (2.3)	1 (1.8)	3 (3.6)	10 (2.2)	
Secondary school leaving certificate/equivalent	11 (8.0)	14 (8.0)	14 (25.0)	15 (18.1)	54 (11.9)	
Specified A-levels	18 (13.0)	17 (9.7)	4 (7.1)	14 (16.9)	53 (11.7)	
A-levels	106 (76.8)	141 (80.1)	37 (66.1)	51 (61.4)	335 (74.0)	
Other	1 (0.7)	0 (0.0)	0 (0.0)	0 (0.0)	1 (0.2)	
**Professional degree *n* (%)**						*Χ*^2^ (21) = 29.82, *p* = 0.096, *C* = 0.25
Currently studying/in vocational training	43 (31.2)	46 (26.1)	20 (35.7)	21 (25.3)	130 (28.7)	
Working without a training degree	1 (0.7)	1 (0.6)	1 (1.8)	5 (6.0)	8 (1.8)	
Vocational training degree	26 (18.8)	34 (19.3)	15 (26.8)	20 (24.1)	95 (21.0)	
Master craftsman/technician/equivalent technical college degree	4 (2.9)	3 (1.7)	1 (1.8)	4 (4.8)	12 (2.6)	
Bachelor’s degree	32 (23.2)	37 (21.0)	7 (12.5)	11 (13.3)	87 (19.2)	
Master’s degree (or equivalent)	28 (20.3)	51 (29.0)	9 (16.1)	21 (25.3)	109 (24.1)	
PhD	3 (2.2)	2 (1.1)	1 (1.8)	0 (0.0)	9 (1.3)	
Other	1 (0.7)	2 (1.1)	2 (3.6)	1 (1.2)	6 (1.3)	
**Current employment**						*Χ*^2^ (3) = 2.99, *p* = 0.394, *C* = 0.08
Yes	96 (69.6)	119 (67.6)	33 (58.9)	60 (72.3)	308 (68.0)	
No	42 (30.4)	57 (32.4)	23 (41.1)	23 (27.7)	145 (32.0)	
**Dermatological diagnoses *n* (%)**						
Acne (yes/no)	0/138 (0/100)	90/86 (51/49)	0/56 (0/100)	55/28 (66/34)	145/308 (32/68)	***Χ***^**2**^**(3) = 165.67, *p* < 0.001, *C* = 0.52**
Atopic dermatitis (yes/no)	0/138 (0/100)	88/88 (50/50)	0/56 (0/100)	33/50 (40/60)	121/332 (27/73)	***Χ***^**2**^**(3) = 126.69, *p* < 0.001, *C* = 0.47**
Psoriasis (yes/no)	0/138 (0/100)	13/163 (7/93)	0/56 (0/100)	8/75 (10/90)	21/432 (5/95)	***Χ***^**2**^**(3) = 17.14, *p* < 0.001, *C* = 0.19**

SH, skin-healthy; DC, dermatological condition only; SP, skin picking only; SP/DC, skin picking and dermatological condition; significant group differences are indicated in bold print.

### 3.2. Group comparisons in outcome variables

The two MANOVAs indicated significant group differences for appearance concerns [*F*(6, 896) = 19.92, Wilks’ *Λ* = 0.78, *p* < 0.001] and mental health variables [*F*(6, 896) = 16.24, Wilks’ *Λ* = 0.81, *p* < 0.001]. The following ANOVAs showed highly significant group differences (all ps < 0.001) with large effect sizes (*η*^2^ > 0.14) for all variables except ARS-D (*η*^2^ = 0.08, medium effect size). In all variables, the SH group had the lowest values (respectively the highest for self-esteem), followed by the DC group. The SP/DC group had the highest values for all variables (and the lowest values for self-esteem), except for anxiety, for which the SP group reported minimally higher values (see Table 2).

**Table 2:** Descriptive statistics and ANOVA-results for the outcome variables.

Variable	SH	DC	SP	SP/DC	Test statistics (ANOVA)
*n*	138	176	56	83	
	***M* (SD)**	***M* (SD)**	***M* (SD)**	***M* (SD)**	
Appearance concerns					
Dysmorphic concerns (DCQ)	6.06 (4.05)	9.06 (4.79)	10.18 (4.79)	12.42 (4.06)	***F*(3, 180.1) = 44.49, *p* < 0.001, *η***^**2**^ **= 0.20**
Appearance-based rejection sensitivity (ARS-D)	11.25 (7.41)	12.40 (7.93)	16.78 (8.66)	17.03 (8.31)	***F*(3, 449) = 13.49, *p* < 0.001, *η***^**2**^ **= 0.08**
Mental health					
Depression (PHQ-9)	5.69 (4.50)	8.35 (6.25)	10.70 (5.96)	12.27 (5.51)	***F*(3, 177.2) = 32.42, *p* < 0.001, *η***^**2**^ **= 0.15**
Anxiety (GAD-7)	5.76 (4.36)	7.72 (5.18)	11.09 (5.02)	11.05 (4.76)	***F*(3, 449) = 28.44, *p* < 0.001, *η***^**2**^ **= 0.16**
Self-esteem					
Self-esteem (RSES)	22.68 (5.75)	20.76 (6.93)	17.25 (6.87)	15.01 (6.90)	***F*(3, 177.4) = 27.81, *p* < 0.001, *η***^**2**^ **= 0.16**
Skin picking					
Skin picking symptoms (mSPS-D)	4.61 (4.09)	10.47 (6.85)	19.63 (5.41)	20.84 (4.63)	***F*(3, 181.1) = 283.21, *p* < 0.001, *η***^**2**^ **= 0.56**

SH, Skin-healthy; DC, Dermatological condition only; SP, Skin picking only; SP/DC, Skin picking and dermatological condition; DCQ, Dysmorphic Concerns Questionnaire; ARS-D, Appearance based Rejection Sensitivity Scale; mSPS-D, modified Skin Picking Scale; PHQ-9, Patient Health Questionnaire-9 for depression; GAD-7, General Anxiety Disorder Scale-7; RSES, Rosenberg Self-Esteem Scale. ANOVAs with corrected degrees of freedom indicate Welch-ANOVAs with homogeneity corrections. Significant test statistics are indicated in bold print.

**Figure 1:**
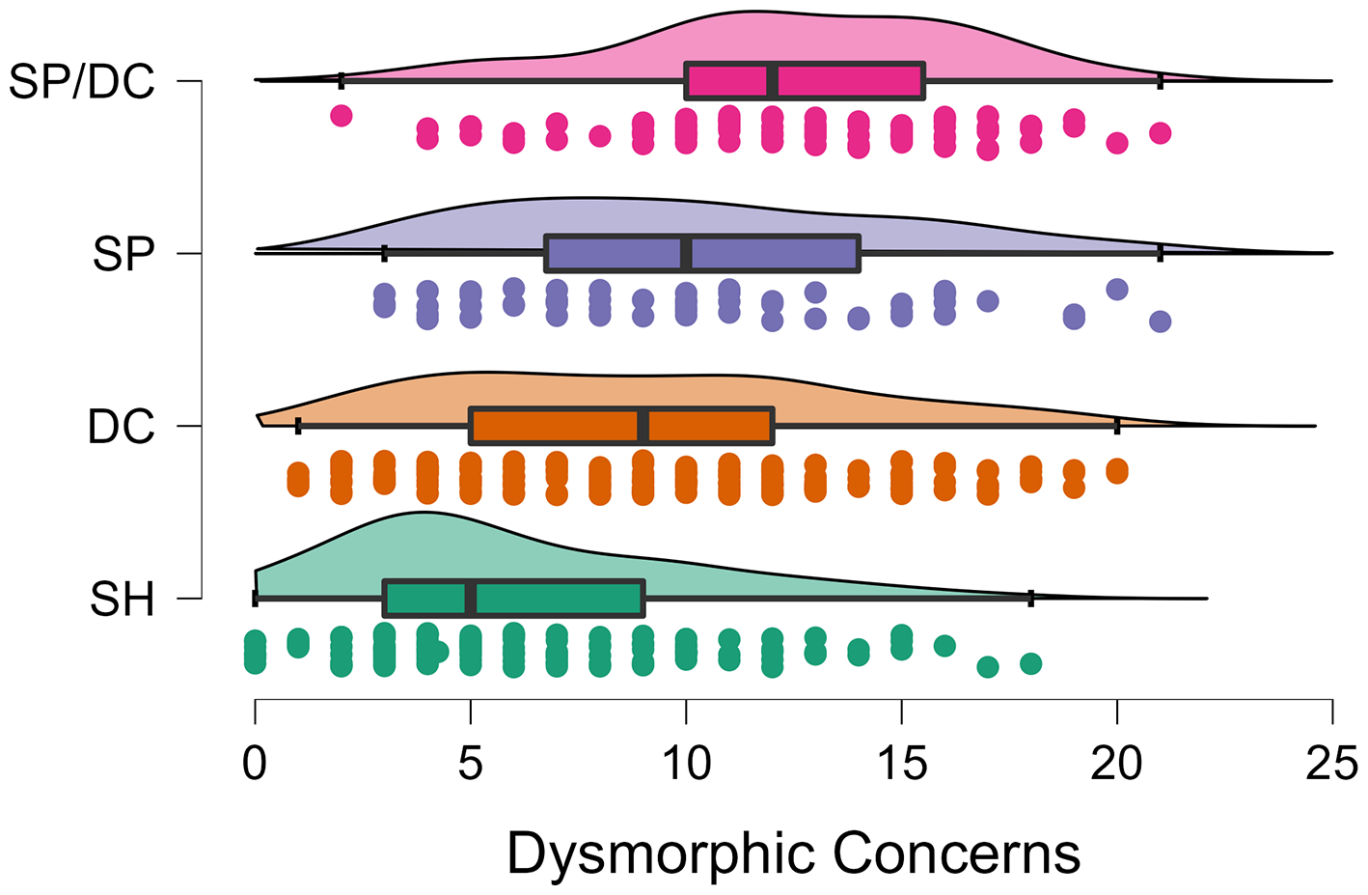
Boxplots of the four subgroups with datapoints and distributions of dysmorphic concerns. SH, skin-healthy; DC, dermatological condition only; SP, skin picking only; SP/DC, skin picking and dermatological condition; scale range of the Dysmorphic Concerns Questionnaire: 0–21.

**Figure 2:**
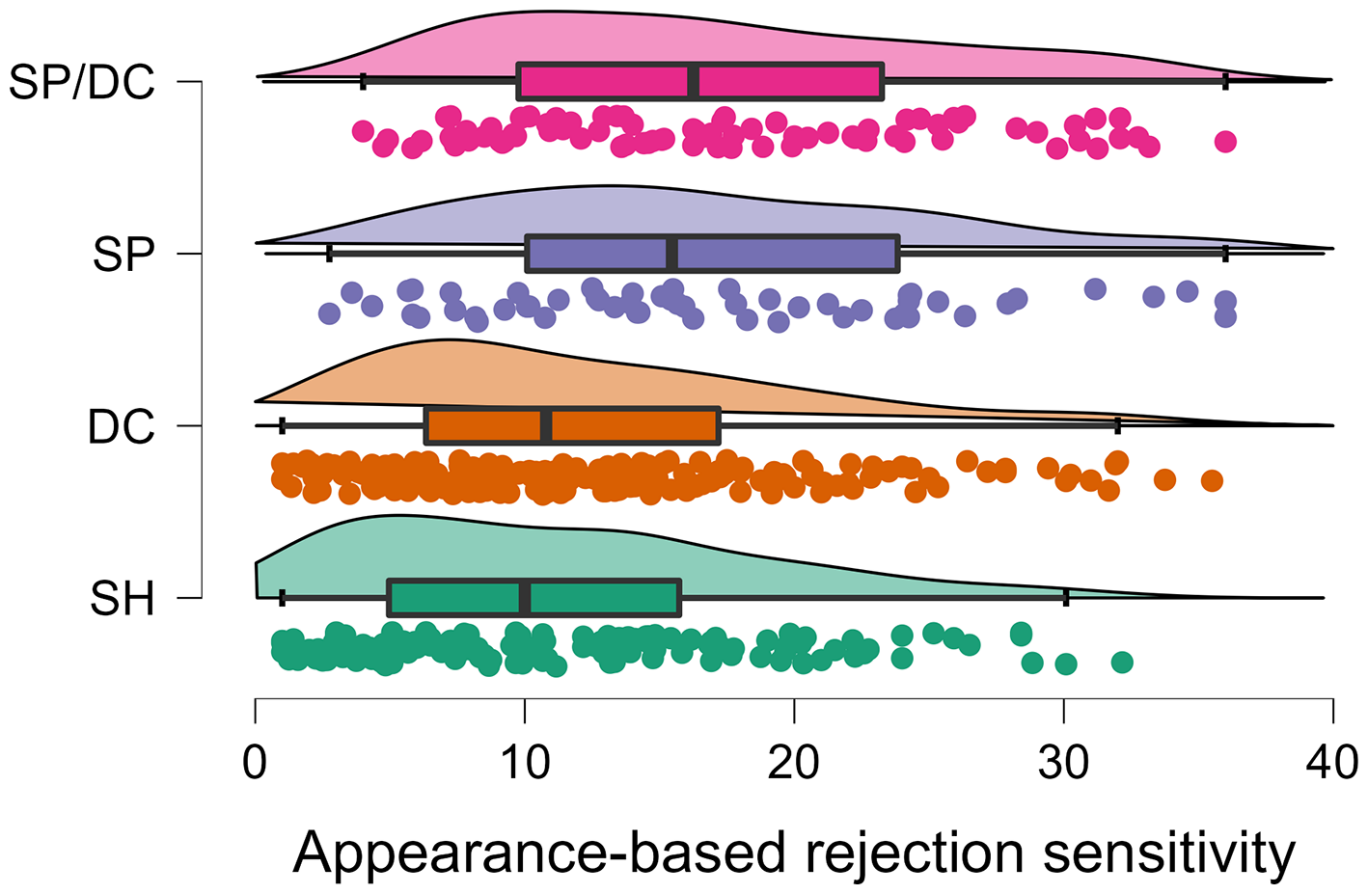
Boxplots of the four subgroups with datapoints and distributions of appearance-based rejection sensitivity. SH, skin-healthy; DC, dermatological condition only; SP, skin picking only; SP/DC, skin picking and dermatological condition; scale range of the Appearance-based Rejection Sensitivity Scale: 0–36.

*Post hoc* group comparisons showed that the subgroups differed significantly in most of the variables. Regarding appearance concerns, the SH group differed significantly from the DC, SP, and SP/DC groups (*p*s < 0.001). However, regarding ARS, there was no significant difference between the SH and DC groups regarding their concerns on appearance-based rejection (*p* > 0.999). In addition, the DC and SP group did not differ significantly in their DCQ-scores indicating comparable dysmorphic concerns (*p* = 0.431). Overall the results of the appearance concerns indicated that the SH group had the lowest concerns, followed by the DC group, the SP group, and the SP/DC group, which differed significantly from the SP group in DCQ-values, but not in the ARS-D score (see Figures 1, 2).

With regard to skin picking assessments, almost all groups differed highly significantly in their skin picking symptoms (mSPS-D), with the SH group showing the lowest scores and significantly lower scores than all other groups (ps < 0.001), followed by the DC group. The scores of both of these groups were significantly exceeded by the two skin picking groups (SP and SP/DC). However, these two groups did not differ significantly among themselves in the assessed skin picking symptoms (*p* = 0.516).

A similar pattern of results was seen in the mental health variables, with smaller differences between groups. With regard to depression, the DC, SP and SP/DC groups all had higher depression scores than the SH group (ps <0.001). The SP group and the DC group did not differ significantly from each other (*p* = 0.060). Further, there were no significant differences between the SP group and the SP/DC group (ps > 0.242). In general, however, the SP/DC group was found to be the most impaired in almost all mental health variables (except GAD-7, where it was nearly equal to the SP group), followed by the SP group, the DC group, and the SH group, which was the least impaired. The results of all *post hoc* comparisons are displayed in Table 3.

**Table 3:** Pairwise group comparisons (*post hoc* tests) in all relevant outcome variables.

Group comparison(*post hoc* test)	SH vs. DC	SH vs. SP	SH vs. SP/DC	DC vs. SP	DC vs. SP/DC	SP vs. SP/DC
DCQ	**<0.001**	**<0.001**	**<0.001**	0.431	**<0.001**	**0.025**
ARS-D	>0.999	**<0.001**	**<0.001**	**0.002**	**<0.001**	>0.999
mSPS-D	**<0.001**	**<0.001**	**<0.001**	**<0.001**	**<0.001**	0.516
PHQ-9	**<0.001**	**<0.001**	**<0.001**	0.060	**<0.001**	0.400
GAD-7	**0.003**	**<0.001**	**<0.001**	**<0.001**	**<0.001**	>0.999
RSES	**0.038**	**<0.001**	**<0.001**	**0.007**	**<0.001**	0.242

SH, skin-healthy; DC, dermatological condition only; SP, skin picking only; SP/DC, skin picking and dermatological condition; DCQ, Dysmorphic Concerns Questionnaire; ARS-D, Appearance Based Rejection Sensitivity Scale; mSPS-D, modified Skin Picking Scale; PHQ-9, Patient Health Questionnaire-9 for depression; GAD-7, General anxiety scale-7; RSES, Rosenberg Self-Esteem Scale. *p*-values for *post hoc* tests are Bonferroni-corrected for simple ANOVAs and Games-Howell *post hoc* comparison are reported for Welch ANOVAs. Significant values are indicated in bold print.

Screening for possible symptoms of BDD showed that the proportion of cases with positive screening was lowest in the SH group at 10 of 138 participants. In the DC group, the proportion of individuals with BDD symptoms was three times higher (38 of 176 participants). Over 40% of individuals in the SP group (25 of 56 participants) showed indications for a positive BDD screening and in the SP/DC group, the proportion of individuals with positive screenings (53 of 83 participants) was almost 64%. Thus, comparable to the other variables for appearance-related concerns, individuals with skin picking are significantly more likely to be affected by BDD symptoms, with individuals with dermatological conditions also showing an increased prevalence. Accordingly, the group difference was highly significant, *χ*^2^(3) = 94.07, *p* < 0.001, *C* = 0.42.

### 3.3. Additional analyses

Given the significant group differences in age and gender, all ANOVAs were repeated as analyses of covariance (ANCOVAs), using age and gender as covariates. Overall, the results did not differ from the reported pattern, except for a nonsignificant difference in self-esteem between the SH and DC group (*p* = 0.076).

## 4. Discussion

The aim of the present study was to investigate the extent to which individuals with PSP differ from control groups with and without dermatological conditions with respect to their appearance-related concerns. Thus, the study intended to extend the evidence on the role of appearance-related aspects in PSP that might contribute to the phenomenology and maintenance of this mental disorder. While earlier research has already shown that body image concerns may play a role in PSP (23) as well as in dermatological conditions [e.g., (30, 31)], specific differences between individuals with PSP, those with dermatological conditions and skin-healthy controls have so far not been analyzed. The present study therefore examined possible group differences in appearance concerns and mental health outcomes between four groups with different skin related impairments [skin-healthy (SH), dermatological conditions only (DC), skin picking only (SP), and a combination of skin picking and dermatological conditions (SP/DC)].

Throughout all variables, appearance concerns, skin picking assessments, and mental health outcomes, we found that individuals with PSP were significantly more affected than individuals with dermatological conditions only or skin-healthy controls. Except for dysmorphic concerns, the PSP groups with and without any diagnosed skin conditions did not differ significantly from each other. Compared to suggested cut-off scores to assess clinically relevant dysmorphic concerns (45, 47), on average both PSP groups exceed the respective cut-off scores and are therefore subjects to a high body image impairment.

Still, the SP/DC group showed the strongest impairments in almost all variables and the highest proportion of possibly clinically relevant symptoms of BDD. Thus, appearance concerns might arise from or be aggravated by existing skin conditions in individuals with PSP. Further, skin conditions can trigger the development and single episodes of PSP (12–14). However, PSP alone seems to account for a marked impairment regarding appearance concerns, such as dysmorphic concerns or appearance-based rejection sensitivity. This result is in line with a recent study by Gallinat et al. (23), who found that skin picking severity was positively and significantly correlated with appearance variables such as body image disturbances and appearance orientation even after controlling for depressive symptoms. In the present study, we were able to replicate this finding. Moreover, we used a broad range of measures to capture different facets of appearance concerns, such as dysmorphic concerns, BDD symptoms and the interpersonal construct of ARS, which has not been investigated in PSP so far. Last, this study adds new knowledge on the potential even aggravating role of dermatological conditions regarding these appearance concerns in individuals with and without PSP.

With regard to dermatological conditions only, we found individuals in the DC group (including participants with diagnosed acne, atopic dermatitis or psoriasis) to be still significantly more affected than skin-healthy controls, regarding their appearance concerns, but not the fear of being rejected due to their appearance. Thus, while possible visible differences might lead to more cognitive concerns, they do not seem to impair the respective individuals that much in their interpersonal relationships. With regard to mental health, slight impairments were visible but the differences to skin-healthy controls were less pronounced than those to the PSP groups. In addition, according to the cut-off scores for the PHQ-9 suggested by Kroenke et al. (56), both, skin-healthy participants and those with dermatological conditions would be classified as reporting mild depressive symptoms (values of 5 to 9), while the PSP groups scored in the range of moderate depressive symptoms (values of 10 to 14). This underlines the distressing nature of PSP which can impair other mental health outcomes.

While this finding is in line with results from previous studies on body image and mental health impairment in dermatological patients (e.g., 30–38), the results of the present study also indicate that skin picking, as a common behavioral pattern in dermatological patients (12, 14), should be given particular sensitive attention. Skin picking can lead to additional appearance-related impairments as well as negatively affect mental health of individuals with skin conditions and should therefore be assessed and addressed by dermatological professionals and, if present, be treated in cooperation with specialists for psychodermatological conditions with primary psychopathology [see, e.g., (57)].

The present study also highlights that, while previous research has mainly focused on skin picking as a behavioral symptom of BDD (24, 25), the co occurrence of both phenomena should be considered in clinical settings. To date, the possible comorbid diagnosis of SPD is often overlooked in patients with BDD who might not exclusively pick their skin to remove blemishes or change their appearance, but also show automatic forms of PSP. Further, the fact that BDD can arise secondarily from the possible visible consequences of skin picking—since the focus is very strongly placed on the skin appearance—has hardly been investigated so far. This cooccurrence of symptoms offers important impulses for practice, especially when it comes to treating SPD in psychological therapies.

While the current evidence-based therapies for SPD rely on cognitive behavioral therapy—mainly cognitive behavior therapy incorporating habit reversal techniques [i.e., (58)]—body image and appearance concerns are still very rarely considered in the treatment approaches for SPD. However, these concerns may be a major contributor to the observed social withdrawal and everyday impairments in SPD (18). The specific approach of addressing appearance-related concerns and body image aspects in therapy, as it is used for example in the therapy of BDD or eating disorders, could therefore significantly enrich the therapy for SPD and reduce psychosocial impairments in this group.

The present study has the strength of comparing groups with and without PSP and dermatological conditions, including a relatively large sample of individuals with PSP via the recruitment support of the large German Self-Help Network for Skin Picking. Further, the results of the group comparisons remain stable even in additional analyses that control for possible influences of gender and age. However, the study is also subject to several limitations.

First, this is an online study in which only psychometrically valid screening instruments for PSP, appearance concerns and for mental health variables were used. However, this cannot replace clinical diagnostics by appropriately trained experts. Therefore, it is important to conduct corresponding studies also in face-to-face settings in mental health and dermatological settings in order to be able to distinguish the groups based on clinical diagnoses by medical experts. This would further allow for more objective assessments of the exaggerated nature of an individual’s appearance-related concerns which are a prerequisite for the clinical diagnosis of BDD. With regard to the inclusion criteria of dermatological conditions, it should additionally be noted that the selection of subjects was based on lifetime diagnoses and not on current complaints. Since even past skin diseases without acute impairment can result in visible and permanent skin changes (e.g., acne scars), we did not exclusively include currently acute complaints. At the same time, however, the screening question targeted existing medical diagnoses. Thus, there is the possibility that persons were excluded from the analysis who suffer from acute skin complaints but do not have a medical diagnosis. Since we limited ourselves to three disorders (acne, atomic dermatitis and psoriasis), which in many cases are frequently medically examined in Germany, we nevertheless assume a good representation of the sample. However, it must be emphasized that some persons were certainly excluded despite existing current skin conditions without medical diagnoses.

Second, the proportion of women in the analysis sample is disproportionately high, especially in the PSP groups. Even though the gender ratio in older studies is very high with a share of 75–94% women in PSP and SPD (59), the percentage of women in our study even exceeds this upper limit. In addition, more recent studies using diagnostic screenings based on DSM-5 criteria have found a more balanced gender ratio in SPD [e.g., (8, 11)]. Furthermore, the gender distribution in the DC groups does not correspond to the usual, more gender-balanced ratios, in larger epidemiological studies on the prevalence of skin conditions [e.g., (60)]. In future studies, more attention should be paid to the recruitment of male patients. In addition, it should be noted that for the present study, participants were in part specifically recruited from corresponding topic forums, Facebook groups and dermatological practices. Therefore, this is not a representative sample and the prevalence found here, both for PSP and for clinically relevant mental health impairments, deviate significantly from the general population, which may also correspond to the proportion of persons with BDD symptoms.

Third, we did not assess appearance concerns specifically targeting aspects of the skin. The DCQ as well as the ARS-D and the BDD screening also assess concerns about other aspects of the body, such as weight or height. Given that there is for example, according to a recent study by Grant and Chamberlain (11) a relatively high comorbidity of SPD and eating disorders, we cannot disentangle, whether the worries of the participants in our study result from their skin conditions (only) or other aspects related to their external appearance. Using additional measures that specifically address concerns and dissatisfaction regarding the skin [e.g., cutaneous body image scales, see (31)] instead of more general appearance-related screening instruments as well as additional questions to rule out possible weight concerns might provide deeper insights and unveil more relevant differences between individuals with PSP compared to dermatological patients.

To shed more light on the potential role of appearance-related concerns in the etiology of PSP and SPD, future studies should implement longitudinal designs to disentangle psychopathological mechanisms. Due to the cross-sectional design, we cannot deduce, whether PSP is the cause or a symptom of appearance-related concerns.

Future research could also include additional experimental studies regarding possible differences in the (tactile or visual) perception of their own body and elicited urges in patients with SPD compared to control groups. For example, in the study of Mehrmann et al. (44) individuals with SPD showed a higher urge to pick their own skin in response to the presentation of visual skin-picking-related stimuli, compared with skin-healthy controls and patients with atopic dermatitis. In an experimental study with functional Magnetic Resonance Imaging, Schienle et al. (61) further demonstrated that patients with SPD, who were confronted with visual images of skin irregularities, reported higher levels of disgust and corresponding specific neural responses (greater activation of the amygdala and insula). Based on the tactile sensory modality, Houghton et al. (62) showed that a mixed group with SPD and Hair Pulling Disorder had a low tactile sensory threshold (i.e., increased tactile sensitivity) compared to a healthy control group, which could account for a different response to skin irregularities.

Such differences in the perception of visual and tactile cues may on the one hand promote PSP symptoms, but on the other hand also cause a different perception of one’s own body and thus also account for appearance-related concerns. However, to date, there are still no distinct comparisons of individuals with PSP to those with different skin conditions regarding self-referential perceptive processes that could further illuminate processes in the formation of appearance concerns and possible treatment approaches.

In addition, intervention studies on cognitive-behavioral therapies or self-help interventions for individuals with PSP should explicitly examine therapeutic components that address body image and appearance-related concerns. This is also especially important for patients with dermatological conditions and PSP, in whom the factor of appearance-related concerns is seldom addressed in health care. Those interventions could aim at changing the importance of appearance for the individual via cognitive restructuring or enable patients to discover new sources of their self-esteem in therapy [e.g., (63)]. This could alleviate the distress and suffering of individuals with PSP and SPD and potentially promote long-term treatment success.

## 5. Conclusion

Overall, we found that appearance concerns constitute an important phenomenological aspect of PSP that has long been neglected and should be further examined and addressed in interventions for individuals with SPD as well as for dermatological patients who exhibit skin picking as a behavioral pattern that could aggravate their skin conditions and cause additional mental health impairments.

## Data availability statement

The raw data supporting the conclusions of this article will be made available by the authors, without undue reservation.

## Ethics statement

The studies involving human participants were reviewed and approved by Ethics Committee of the University of Wuppertal. The patients/participants provided their written informed consent to participate in this study.

## Author contributions

JS and AM: conception and design of the study. JS, CG, and AM: interpretation of the data, drafted the work, and revised the paper. All authors contributed to the article and approved the submitted version.

## Funding

The library of the FH Münster University of Applied Sciences contributed to the open access publication fees.

## Conflict of interest

JS declares that she has received a financial compensation by the German Self-Help Network for Skin Picking to present the findings of the study on the German Self-Help Meeting “BFRB Tage 2021” to present the results of this study.

The remaining authors declare that the research was conducted in the absence of any commercial or financial relationships that could be construed as a potential conflict of interest.

## Publisher’s note

All claims expressed in this article are solely those of the authors and do not necessarily represent those of their affiliated organizations, or those of the publisher, the editors and the reviewers. Any product that may be evaluated in this article, or claim that may be made by its manufacturer, is not guaranteed or endorsed by the publisher.
